# Cortical asymmetry and its developmental change have distinct relationships with brain connectivity

**DOI:** 10.64898/2026.10.28.762579

**Published:** 2026-10-28

**Authors:** Siva Venkadesh, Fang-Cheng Yeh, Patrick R. Hof, Julie R. Korenberg

## Abstract

The two cerebral hemispheres specialize while remaining interconnected. Does greater structural asymmetry imply less connectivity, and does connectivity also relate to how asymmetry changes during development? We addressed these questions in three independent cohorts spanning birth to 22 years, measuring asymmetry as the magnitude of the left–right difference in association tract overlap across 74 homotopic cortical region pairs. The regional pattern of asymmetry was similar across cohorts. In all three cohorts, more asymmetric regions had lower connectional density to cortical and non-cortical targets. However, involvement of the corpus callosum, the major pathway connecting the hemispheres, showed no consistent inverse relationship with regional asymmetry across cohorts. Instead, callosal involvement conditioned the relationship between mean regional asymmetry and its age-related change. In the two older cohorts, greater mean asymmetry accompanied more positive age effects among regions with appreciable callosal overlap, whereas this relationship was weak or negative among regions with little callosal overlap (standardized interactions +0.42 and +0.56). The pattern replicated across the two cohorts at matched ages, and longitudinal region-mean estimates in the Adolescent Brain Cognitive Development cohort were similar in direction and magnitude. In the youngest cohort, regions converged toward the mean, narrowing the between-region spread by 12%, with no detected callosal moderation. More asymmetric regions have lower overall connectional density, whereas callosal involvement helps explain how regional asymmetry changes with age.

**Significance statement:**

The brain’s hemispheres differ in structure and function while remaining extensively connected. Does connectivity relate to how asymmetric a region is, how its asymmetry develops, or both? We distinguish these questions in three developmental cohorts. More asymmetric regions had lower overall connectional density. In contrast, involvement of the corpus callosum, the major pathway linking the hemispheres, showed no consistent inverse relationship with asymmetry across cohorts. Instead, it distinguished how mean regional asymmetry related to age-related change in the two older cohorts. A static comparison would miss this developmental relationship. These findings provide a framework for studying hemispheric development and investigating whether connectivity–asymmetry relationships are altered in neurodevelopmental conditions.

## Full Text Availability

The license terms selected by the author(s) for this preprint version do not permit archiving in PMC. The full text is available from the preprint server.

